# Iron deficiency and protection of blood donors

**DOI:** 10.1590/S1516-31802001000400002

**Published:** 2001-07-07

Modern medicine should not discard the advances in blood transfusion. Day by day, new and more intensive protocols are being developed in order to increase survival and cure rates in patients, especially those affected by cancer. Moreover, genetic diseases such as Hemophilia A or B, urban violence and traffic accidents have increased the needs related to whole blood and its components and industrially derived products.

Obtaining blood supplies of sufficient quality and quantity while preserving the health of donors is an important challenge for the Blood Transfusion Institutions. Increasing investments have been made in serology control in order to promote recipient protection, and this is at present the main focus of donor screening. The Brazilian rules and laws are very strict in relation to serological control, and penalties are laid down for professionals and Institutions that do not carry out the determinations.

Clinical aspects like those showed in the paper "Iron Deficiency in Blood Donors" (São Paulo Med J/Rev Paul Med 2001;119(4):132-4) are rarely studied and presented in our country, in spite of the high frequency of anemia in our population and the development of an important public Blood Transfusion System. Iron deficiency is very common worldwide, especially in developing countries. In general, Brazilian blood donors are poor people, who have low income and non-standard life conditions.

Several papers have shown that depletion of iron reserves and iron dependent anemia are more frequent among blood donors, particularly among females and multi-time donors. It is known that multi-time donors are the safest blood donors. Frequent donation, performing frequent serological screening, reduces the probability of infectious diseases being transmitted by blood.

The major reason for determining the donor's hemoglobin concentration prior to donation is to ensure either that the donor does not have pre-existing anemia or that the donor will not be made anemic by the blood donation. Iron deficiency may exist in the absence of overt anemia. Over the past decade, several studies have attempted to assess the appropriate minimum hemoglobin levels by determining the ability of proposed cutoffs to detect donors with iron deficiency in the absence of overt anemia. Unfortunately, all the proposed minimum hemoglobin levels are poorly predictive of body iron storage.

If the intent is to ensure that donors do not become iron depleted, it will be necessary to perform a more direct test for measurement of iron metabolism, such as serum ferritin, which is not readily available due to the complexity and expense of the assay. Additional attention to quality assurance of donor screening procedures is warranted due to the situations that exist in some blood transfusion settings.

